# Building a cognitive map through self-motion

**DOI:** 10.7554/eLife.104500

**Published:** 2024-11-25

**Authors:** Bharath Krishnan, Noah Cowan

**Affiliations:** 1 Department of Biomedical Engineering, Johns Hopkins University Baltimore United States; 2 Zanvyl Krieger Mind/Brain Institute, Johns Hopkins University Baltimore United States; 3 Kavli Neuroscience Discovery Institute (NDI), Johns Hopkins University Baltimore United States

**Keywords:** spatial learning, cognitive map, shortcut, open maze, navigation, trajectory analysis, Mouse

## Abstract

Mice can generate a cognitive map of an environment based on self-motion signals when there is a fixed association between their starting point and the location of their goal.

**Related research article** Xu J, Girardi-Schappo M, Beique JC, Longtin A, Maler L. 2024. Shortcutting from self-motion signals reveals a cognitive map in mice. *eLife* **13**:RP95764. doi: 10.7554/eLife.95764.

When moving through an environment, we often use visual landmarks – such as a specific store or street sign – to guide us and determine our next action (Tolman et al., 1946a). However, the brain does not just rely on visual landmarks for navigation. It also performs path integration, a process that uses self-motion signals – such as velocity and acceleration – to estimate our position in an environment relative to where we started (Mittelstaedt and Mittelstaedt, 1980; Wittlinger et al., 2006; Savelli and Knierim, 2019; Etienne and Jeffery, 2004). This ability is why you can walk through a dark room and still maintain a sense of your location.

In mammals, path integration can also update an internal estimate of position on a ‘cognitive map’, a neural representation of a known environment containing information on the distances, directions and spatial relationships between locations. However, how cognitive maps initially form, and the amount and type of information that is required to build them, is not fully understood. Now, in eLife, Leonard Maler and colleagues from the University of Ottawa – including Jiayun Xu and Mauricio Girardi-Schappo as joint first authors – report that mice can create cognitive maps by relying predominantly on path integration (Xu et al., 2024).

The team designed a clever experimental apparatus called the Hidden Food Maze, which contains 100 holes where food can be concealed. Around the edge of the circular maze are four entrances spaced 90 degrees apart, creating four quadrants. This layout results in locations that are ‘rotationally equivalent’, meaning each quadrant has locations that correspond to sites in the other three quadrants. External visual cues are also displayed on the walls of the arena, which the mice could potentially use to navigate through the maze.

The mice were trained over multiple trials to find food hidden in one of the maze’s 100 holes. In random entrance experiments, the mice entered the maze through a different door for each trial, whereas in static entrance experiments, they entered through the same door each time. Xu, Girardi-Schappo et al. observed that when the mice used different entrances, they were unable to learn the location of the food (Figure 1A). However, when mice entered the maze through the same entrance each time, they quickly learned where the food reward was hidden (Figure 1B). This was demonstrated by mice that had been trained in the static entrance experiment taking a shorter, more direct route and checking more holes near the food location.

**Figure 1.**
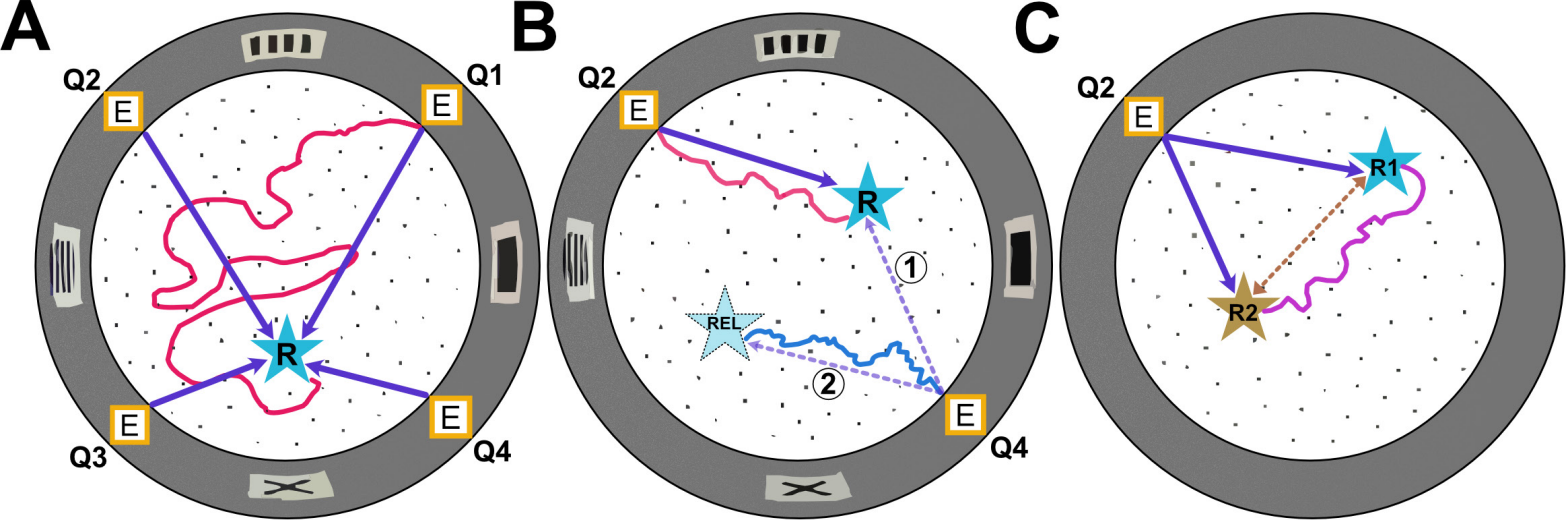
Types of experiments in the Hidden Food Maze. The Hidden Food Maze developed by Xu, Girardi-Schappo et al. is a circular arena equipped with four evenly spaced entrances (marked E), multiple holes for concealing food (black dots), and various visual landmarks (grey rectangles) displayed on its walls. (**A**) In the random entrance experiment, the mice entered the maze through a different door in each trial to find a food reward (R, blue star) that was always located in the same place. The dark purple arrows represent the most direct route the mice could take from each entrance. However, despite significant training, the mice failed to learn where the food was concealed as illustrated by the red line which represents a hypothetical trajectory a mouse may have taken. (**B**) In the static entrance experiment, the mice always entered through the same entrance (marked Q2). With training, the mice quickly learned how to reach the food (hypothetical red trajectory), taking a direct route to the reward from entrance Q2 (dark purple arrow). Following training, a probe trial was introduced in which the mice entered the maze via a different door (Q4). There were two logical routes (purple dashed arrows) that the mice could have taken in probe trials. If the mice relied primarily on visual landmarks, they would have taken route 1, a direct route to the correct food location. However, if they relied on path integration, they would take route 2, the rotationally equivalent location. Surprisingly, in probe trials, the mice ignored visual landmarks and navigated to the rotationally equivalent location (hypothetical blue trajectory). (**C**) In the two-food location experiment, mice were sequentially trained to locate food at two different sites (R1 and R2). During probe trials where no food was present at either location, the mice took a novel direct shortcut (hypothetical purple trajectory) between R1 and R2 (dashed brown line), indicating that they had formed an internal cognitive map of the spatial relationship between the two reward sites.

Image credit: Adapted from the paper by Xu et al., 2024.

Once the mice learned the location of the food in the static entrance experiment, the team conducted probe trials where the mice entered the maze through a different door. In these trials, the mice consistently navigated to the hole that was rotationally equivalent to the site where the food was originally kept rather than to the actual reward location (Figure 1B). This behavior indicated that the mice ignored the visual landmarks and instead employed another strategy, such as a learned motor sequence or path integration, using their original starting point as a reference location.

Why were the mice not relying on the visual landmarks to navigate? One possibility is that the landmarks used in the task were not striking enough. Alternatively, the mice may have perceived the visual cues as unreliable (Biegler and Morris, 1993; Knierim et al., 1995; Jeffery, 1998), given that the landmarks were absent when the animals were initially familiarized with the environment, and appeared in different locations relative to the animal’s starting location every time they entered through a different door in the random entrance experiment. Nevertheless, these experiments suggest that mice can develop an internal cognitive map based primarily on path integration. Although it is possible that the mice were instead employing a simpler strategy, such as memorizing a sequence of motor actions.

To investigate if the mice were truly using path integration, and not a learned sequence of motor actions, Xu, Girardi-Schappo et al. conducted a third test that they called the two-food location experiment. During the experiment, mice were first trained to find food at one location (R1), and then trained again with the food in a second location (R2; Figure 1C). Once the animals were fully trained on location R2, probe trials were introduced where neither food site contained food. After navigating to R2 and finding it empty, the mice decided to explore their old feeding location, R1. Rather than going back to the home base and venturing out to R1 (which could be done based on a learned motor sequence), they took a novel shortcut directly from R2 to R1. The ability to take novel shortcuts in an environment has long been considered strong evidence for the formation of a cognitive map (Tolman et al., 1946b).

These findings demonstrate that mice can build a cognitive map using self-motion cues alone, without relying on any external landmarks, as long as the spatial relationship between the start and reward locations remains consistent across trials. This kind of learning has previously been observed in humans (Etienne and Jeffery, 2004; Landau et al., 1984). Future experiments using the Hidden Food Maze apparatus could add to the rich literature on the neurophysiological mechanisms underpinning path integration and navigation (Savelli and Knierim, 2019; McNaughton et al., 2006; Madhav et al., 2024), providing new insights into how cognitive maps are influenced by self-motion signals.
